# The Course of Carotid Plaque Vulnerability Assessed by Advanced Neurosonology

**DOI:** 10.3389/fneur.2021.702657

**Published:** 2021-08-20

**Authors:** Sander Johan Aarli, Lars Thomassen, Ulrike Waje-Andreassen, Nicola Logallo, Christopher Elnan Kvistad, Halvor Næss, Annette Fromm

**Affiliations:** ^1^Department of Neurology, Haukeland University Hospital, Bergen, Norway; ^2^Department of Clinical Medicine, University of Bergen, Bergen, Norway; ^3^Department of Biological and Medical Psychology, University of Bergen, Bergen, Norway; ^4^Department of Neurosurgery, Haukeland University Hospital, Bergen, Norway; ^5^SESAM – Centre for Age-Related Medicine, Stavanger University Hospital, Stavanger, Norway

**Keywords:** atherosclerosis, carotid artery disease, contrast-enhanced ultrasound, microembolic signals, neovascularization, plaque vulnerability, Sonazoid

## Abstract

**Background:** Carotid artery atherosclerosis is a major risk factor for ischemic stroke. This risk is related to plaque vulnerability and is characterized by plaque morphology, intraplaque neovascularization, and cerebral microembolization. Advanced neurosonology can identify vulnerable plaques and aid in preventing subsequent stroke. We aimed to assess the time course of cerebral microembolization and intraplaque neovascularization during 6 months of follow-up and to explore the utility of advanced neurosonology in patients with acute cerebral ischemia.

**Methods:** Fifteen patients with acute cerebral ischemia and carotid artery plaques underwent comprehensive extra- and intracranial ultrasound examinations, including microemboli detection and contrast-enhanced ultrasound. The examinations were repeated after 3 and 6 months.

**Results:** We examined 28 plaques in 15 patients. The ultrasonographic features of plaque vulnerability were frequent in symptomatic and asymptomatic plaques. There were no significant differences in stenosis degree, plaque composition, plaque surface, neovascularization, or cerebral microembolization between symptomatic and asymptomatic plaques, but symptomatic plaques had a higher number of vulnerable features. None of the patients had recurrent clinical stroke or transient ischemic attack during the follow-up period. We observed a decrease in cerebral microembolization at 6 months, but no significant change in intraplaque neovascularization.

**Conclusions:** In patients with acute cerebral ischemia and carotid artery plaques, cerebral microembolization decreased during 6 months of follow-up, indicating plaque stabilization.

**Clinical Trial Registration:**ClinicalTrial.gov, identifier NCT02759653.

## Introduction

Carotid artery atherosclerosis is a frequent cause of ischemic stroke (1). Risk stratification and treatment recommendations have traditionally been based on the degree of stenosis and the occurrence of ischemic events in the corresponding vascular territory (2, 3). However, there is evidence that other plaque features also influence the risk of stroke (4–6). This evidence has led to the concept of the vulnerable plaque, characterized by plaque echolucency, lipid-rich necrotic core, thin fibrous cap, infiltration of inflammatory cells, intraplaque neovascularization, intraplaque hemorrhage, surface ulceration, and cerebral microembolization (7–9). Intraplaque neovascularization (IPN) seems to be a useful indicator of plaque vulnerability, and previous studies have shown a strong correlation between the degree of IPN and the risk of plaque rupture and subsequent stroke (10, 11). IPN can be detected and measured *in vivo* by contrast-enhanced ultrasound (CEUS), which corresponds well with histopathological findings (12–14). The presence of microembolic signals (MES) on transcranial Doppler indicates ongoing embolization and is an independent predictor of future stroke in symptomatic and asymptomatic carotid artery disease (15, 16). Data on the time course of MES and IPN in patients with carotid plaques are limited (17–19). We aimed to assess temporal changes in microembolization and neovascularization during a 6-month follow-up study and explored the utility of advanced neurosonology in acute ischemic stroke.

## Materials and Methods

### Study Protocol

The study was conducted at the Department of Neurology at Haukeland University Hospital in Bergen, Norway. The Regional Committee for Medical and Health Research Ethics of Western Norway (REC West 2015/1217) approved the study, and written informed consent was obtained from all participants. We included 15 non-consecutive patients admitted between August 2016 and November 2017 with acute cerebral ischemia and carotid artery atherosclerosis detected on routine duplex ultrasound. Both symptomatic and asymptomatic carotid plaques were included. A carotid artery plaque was defined as symptomatic if it was related to a recent clinical event such as an acute cerebral infarction or a transient ischemic attack (TIA). All patients underwent cerebral computed tomography (CT) and computed tomography angiography (CTA) of the pre- and intracerebral vessels on admission and cerebral magnetic resonance imaging (MRI) on the same or next day. We used a standardized questionnaire from The Norwegian Stroke in the Young Study (NOR-SYS) adapted to our study that comprised medical history, vascular risk factors, and current medication (20). An experienced vascular neurologist (AF) performed all extra- and intracranial ultrasound examinations using a Philips iU22 ultrasound machine with a 9–3 MHz linear array transducer and a 5–1 MHz sector array probe (Philips Medical Systems, Bothell, WA, USA) and a Nicolet^®^ SONARA^®^ Transcranial Doppler System (Natus Neurology Incorporated, Middleton, WI, USA). The non-contrast examinations were performed as described in the NOR-SYS protocol (20). We used the maximum percentage area reduction in the cross-sectional plane for stenosis grading, and the results were stratified as <50, 50–69, ≥70% but less than near occlusion, near occlusion, or total occlusion. Because diameter reduction is an inaccurate parameter in irregular luminal narrowing (21), we chose to use area reduction, as this is a more accurate parameter for the hemodynamic effect of any plaque. Plaque surface was classified as regular, irregular, or ulcerating, and plaque composition was classified as hyperechoic, iso-/hypoechoic, or mixed. IPN was investigated with CEUS using an intravenous bolus injection of 0.3 mL stabilized perfluorobutane (PFB) microbubbles (Sonazoid™, GE Healthcare, Oslo, Norway) as the contrast agent (18, 22). We used a low mechanical index (0.06) to prevent disruption of microbubbles. IPN was graded semi-quantitatively according to previous studies as none, limited (limited appearance of contrast agent bubbles within the plaque), moderate (constant appearance of contrast agent bubbles within the plaque), or extensive (pulsating vessel within the plaque) (23). Microemboli detection was performed before CEUS to avoid misinterpreting PFB microbubbles as stroke-related microemboli. Both middle cerebral arteries were insonated through the temporal bone window with 2 MHz pulsed-wave transducers for 60 min. Power and gain were kept as low as possible to reduce background noise. High-intensity transient signals were automatically detected by the software and independently categorized by two experienced observers (AF and SJA) as either MES or artifacts. We used the MES criteria defined by the Consensus Committee of the Ninth International Cerebral Hemodynamic Symposium (24). In case of discrepancy between the investigators, a consensus was reached through a second joint review. The evaluation of MES was blinded to other plaque characteristics. When assessing the number of vulnerable neurosonological features for each plaque, the degree of stenosis was dichotomized into <70 or ≥70%; plaque surface, into regular or irregular/ulcerating; plaque composition, into hyperechoic or isoechoic/hypoechoic/mixed; and IPN, into non/limited or moderate/extensive. Electrocardiogram and 24-h Holter monitoring were performed to identify possible competing sources of microembolization. Treatment decisions were made according to department routines and clinical guidelines. Medical treatment involved single- or dual antiplatelet therapy combined with high-dose statin therapy and adequate treatment of vascular risk factors. Carotid endarterectomy was considered for patients with symptomatic carotid stenosis >70% irrespective of features of plaque vulnerability, and for patients with symptomatic 50–69% stenosis with features of plaque vulnerability. After 3 and 6 months, outpatient follow-up examinations were performed, including questionnaires regarding recurrent ischemic events, vascular risk factors and adherence to medical treatment, carotid duplex sonography, microemboli detection, and CEUS. The primary outcome measure was recurrent ischemic stroke or TIA. Secondary outcome measures were other acute cardiovascular events (coronary artery disease or peripheral artery disease).

### Statistical Analysis

STATA 16.1 (StataCorp, College Station, TX, USA) was used for statistical analyses. Continuous variables are presented as median (interquartile range) and categorical variables as numbers (percentages). Univariate logistic regression analyses were performed to evaluate whether ultrasonographic features of plaque vulnerability could predict symptomatic plaques at baseline and explore potential associations between the different plaque features. For these analyses, the vulnerable plaque features were dichotomized as previously described. Linear regression was applied to determine whether the number of vulnerable plaque features differed between symptomatic and asymptomatic plaques. We used clustering to adjust for dependency between different plaques in the same patient. To investigate changes in IPN and MES occurrence during follow-up, we fitted a multilevel mixed-effects logistic regression model and adjusted for dependency between repeated measurements in the same plaque and between different plaques in the same patient. In this model, the degree of IPN was dichotomized to none/limited or moderate/extensive in accordance with previous studies (25, 26). Operated plaques were not included in the longitudinal analyses. For all analyses, *p* < 0.05 was considered to indicate statistical significance. Due to the descriptive study design, power calculations were not performed.

## Results

### Patient Demographics

Fifteen patients (age range: 44–70 years) were included. Of these, 13 had bilateral carotid plaques; therefore, 28 plaques were examined in total. Patient characteristics at baseline are presented in Table 1, and ultrasonographic plaque characteristics at baseline are shown in Table 2. The included patients had a considerable burden of vascular risk factors such as hypertension (53.5%), dyslipidemia (46.7%), and current smoking (60%). Further, 60% had a family history of cardiovascular disease, and 66.7% had increased waist-hip ratio and body mass index ≥25 kg/m^2^. The median number of vascular risk factors was 3 (interquartile range: 2–5). Eleven patients had MRI-verified acute cerebral infarctions with corresponding carotid artery plaques. Among the remaining four patients, two had clinical TIA from the anterior circulation with corresponding carotid plaques, one had a branch retinal artery occlusion with corresponding carotid plaque, and one had cerebral infarctions in the posterior circulation only without a fetal origin of the posterior cerebral arteries. None of the acute cerebral infarctions had a lacunar appearance on MRI. Atrial fibrillation or other competing sources of emboli were not detected in any of the patients. Thus, acute cerebral infarctions were considered to be related to the corresponding carotid plaques.

**Table 1:** Patient characteristics at baseline.

	***N*** **= 15**
Age (years)	59.5 ± 8.1
Male sex	11 (73.3)
Previous stroke/TIA	4 (26.7)
Coronary artery disease	2 (13.3)
Peripheral artery disease	1 (6.7)
Hypertension	8 (53.3)
Dyslipidemia	7 (46.7)
Diabetes mellitus	1 (6.7)
Smoking	
Current	9 (60.0)
Former	5 (33.3)
Family history of CVD	9 (60.0)
Overweight/obesity	10 (66.7)
Increased waist-hip ratio	10 (66.7)
Bilateral plaques	13 (86.7)
Bilateral symptomatic plaques	1 (6.7)

*TIA, transient ischemic attack; CVD, cardiovascular disease*.

**Table 2:** Plaque characteristics at baseline.

	**Symptomatic** ***N*** **= 15**	**Asymptomatic** ***N*** **= 13**	**Total** ***N*** **= 28**
**Stenosis degree**			
<50%	7 (46.7)	8 (61.5)	15 (53.6)
50–69%	3 (20.0)	3 (23.1)	6 (21.4)
≥70%	2 (13.3)	2 (15.4)	4 (14.3)
Near occlusion	2 (13.3)	0 (0.0)	2 (7.1)
Total occlusion	1 (6.7)	0 (0.0)	1 (3.6)
**Plaque surface**			
Regular	2 (13.3)	6 (46.2)	8 (28.6)
Irregular	6 (40.0)	2 (15.4)	8 (28.6)
Ulcerating	6 (40.0)	5 (38.5)	11 (39.3)
N/A	1 (6.7)*	0 (0.0)	1 (3.6)
**Plaque composition**			
Hyperechoic	2 (13.3)	4 (30.8)	6 (21.4)
Iso-/hypoechoic	4 (26.7)	1 (7.7)	5 (17.9)
Mixed	9 (60.0)	8 (61.5)	17 (60.7)
**Neovascularization**			
None	2 (13.3)	7 (53.8)	9 (32.1)
Limited	4 (26.7)	3 (23.1)	7 (25.0)
Moderate	6 (40.0)	1 (7.7)	7 (25.0)
Extensive	3 (20.0)	2 (15.4)	5 (17.9)
**Microembolization**			
MES negative	9 (60.0)	6 (46.2)	15 (53.6)
MES positive	5 (33.3)	5 (38.5)	10 (35.7)
Artifacts/not performed	1 (6.7)	2 (15.4)	3 (10.7)
**Number of vulnerable plaque features**			
0	0 (0.0)	2 (15.4)	2 (7.1)
1	0 (0.0)	2 (15.4)	2 (7.1)
2	4 (26.7)	4 (30.8)	8 (28.6)
3	8 (53.3)	4 (30.8)	12 (42.9)
4	3 (20.0)	1 (7.7)	4 (14.3)
5	0 (0.0)	0 (0.0)	0 (0.0)

* *Total occlusion*.

*MES, microembolic signals*.

### Features of Plaque Vulnerability at Baseline

Features associated with plaque vulnerability were frequently found in our study. Twenty-two (78.6%) plaques had iso-/hypoechoic or mixed composition, and 19 (67.9%) had an irregular or ulcerated surface. IPN was found in 19 (67.9%) plaques, and 10 (35.7%) of the plaques had MES in the corresponding vascular territory. The interrater reliability for MES evaluation was substantial, with a kappa score (AF and SJA) of 0.8. Degree of stenosis, plaque composition, plaque surface, degree of IPN, and presence of MES were not significantly different between symptomatic and asymptomatic plaques, but symptomatic plaques had a higher number of vulnerable features (correlation coefficient: 0.93, 95% CI: 0.03–1.83, *p* = 0.04). We found no significant association between the degree of IPN and the occurrence of corresponding MES. The only total occlusion was an acute thrombotic occlusion at the site of a highly neovascularized atherosclerotic plaque with severe luminal narrowing. Repeated CEUS examinations revealed a minimal recanalization that remained non-visualized on CTA.

### Follow-Up

**Figure 1:**
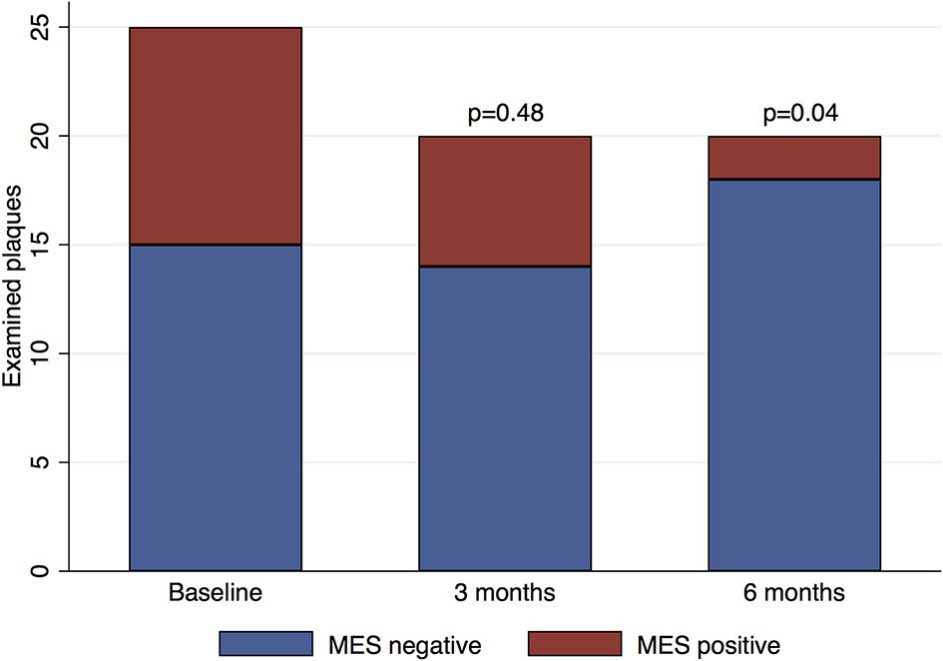
Microemboli occurrence during follow-up. Baseline: 25 examined plaques in 14 patients. Three-month follow-up: 20 plaques in 14 patients. Six-month follow-up: 20 plaques in 13 patients. The surgically removed plaques were excluded from the longitudinal analyses. MES, microembolic signals.

None of the patients reported recurrent clinical stroke or TIA during the follow-up. Further, there were no other acute cardiovascular events in the same period. One patient died of an unknown cause before the 6-months follow-up visit, while the remaining 14 completed the follow-up period according to the study protocol. Six patients underwent unilateral carotid endarterectomy of a symptomatic carotid artery plaque, while the remaining patients received medical treatment. Changes in the degree of IPN and MES occurrence during follow-up are presented in Table 3 and Figure 1. At 3 months, there were no significant changes in the degree of IPN or the occurrence of MES. At 6 months, we found a significant decrease in MES occurrence (OR: 0.15, 95% CI: 0.02–0.90, *p* = 0.04), but the degree of IPN was not significantly different. Surgically removed plaques were excluded from the follow-up analyses.

**Table 3:** Neovascularization and microembolization during follow-up.

	**Baseline** ***N*** **= 28**	**3 months** ***N*** **= 28**	**6 months** ***N*** **= 26**
**Neovascularization**			
None/Limited	16 (57.1)	14 (50.0)	16 (61.5)
None	9 (32.1)	7 (25.0)	9 (34.6)
Limited	7 (25.0)	7 (25.0)	7 (26.9)
Moderate/extensive	12 (42.9)	4 (14.3)	3 (11.5)
Moderate	7 (25.0)	2 (7.1)	2 (7.7)
Extensive	5 (17.9)	2 (7.1)	1 (3.8)
Not performed	0 (0.0)	10 (35.7)	7 (26.9)
**Microembolization**			
MES negative	15 (53.6)	14 (50.0)	18 (69.2)
MES positive	10 (35.7)	6 (21.4)	2 (7.7)
Artifacts/not performed	3 (10.7)	8 (28.6)	6 (23.1)

*MES, microembolic signals*.

## Discussion

Ultrasonographic features of plaque vulnerability were frequent in both symptomatic and asymptomatic carotid artery plaques. The frequencies of plaque echolucency, IPN, and MES occurrence were in the same range as reported in other studies (25, 27, 28). However, comparison between studies was difficult due to methodological differences, heterogeneity in clinical cohorts, and small sample sizes.

We found a significant association between an increasing number of vulnerable plaque features and symptomatic plaques. This finding is expected, as the individual features included are known to predict symptomatic disease (4, 6, 16). In our study, the individual plaque features did not differ significantly between symptomatic and asymptomatic plaques. This may be because of the small sample size, but it may also be related to the fact that the symptomatic and asymptomatic plaques were investigated in the same patients. Atherosclerosis is a systemic disease, and ultrasound features associated with plaque vulnerability were also frequent in asymptomatic plaques. Thus, it may have been a certain degree of coincidence related to whether the patient's right or left carotid plaque became symptomatic first, leading to hospital admission. This concern could have been addressed by including a control group with asymptomatic plaques only, but this was not done in our study.

We did not find an association between increased IPN and MES occurrence, likely because MES detection was done 3–5 days after admission and after the initiation of platelet inhibition. While changes to the degree of IPN are gradual, MES are most frequent in the early phase of acute stroke, and MES occurrence is, to a greater extent than IPN, affected by antithrombotic treatment (29, 30). Other studies have shown an association between IPN and MES occurrence (28, 31). Zhou et al. carried out MES detection within 3 days, while the time frame was not specified in the paper by Ritter et al. Both studies used sulfur hexafluoride (SonoVue^®^) as the contrast agent. We chose to use PFB microbubbles because PFB showed better contrast properties with higher signal intensity and more extended durability than sulfur hexafluoride, in our experience.

We observed a significant decrease in MES occurrence at 6 months. To our knowledge, no other study has examined the course of MES in the months after a stroke or TIA, but one study found a similar decrease in MES occurrence at 1 year compared to the baseline examination (17). This decrease is plausible and probably attributed to antithrombotic and statin treatment and the elapsed time since the ischemic event, indicating disease stabilization. The decreased MES occurrence in our study also corresponds to the absence of recurrent clinical stroke or TIA during follow-up.

Histopathological studies have found an inverse correlation between the elapsed time after an ischemic event and the degree of IPN, implying a decrease in IPN during plaque remodeling (18, 32). During the 6 months of follow-up, we found no significant changes in IPN. A Japanese CEUS study found a non-significant trend toward reduced IPN in plaques that had been asymptomatic for >6 months (18), while another study demonstrated reduced IPN after 2 years of atorvastatin therapy (19). It is possible that 6 months of follow-up is insufficient to detect a decrease in IPN, as these changes are gradual. The semi-quantitative grading of IPN used in our study may be less precise than a quantitative approach. Commercially available software enables quantitative determination of IPN, but the current software programs have limitations with respect to movement artifacts, calcified plaques, and pseudo-enhancement in the far wall due to non-linear propagation of ultrasound waves (14, 33). Most of the plaques in our study were located in the far wall of the carotid vessel, and we considered a semi-quantitative grading to be satisfactory. A comparative study shows a good correlation between semi-quantitative grading and quantitative measurements (34).

Due to the absence of recurrent ischemic events, it is not possible to determine the prognostic value of neurosonological surrogate markers of plaque vulnerability. However, our longitudinal data support the use of advanced neurosonology to observe plaque stabilization over time and to identify plaques that remain vulnerable and may need intensified medical treatment or surgical intervention. To our knowledge, this is the first study to assess the course of both cerebral microembolization and intraplaque neovascularization in the months following an ischemic event attributed to carotid artery disease. The main strengths of our study are the comprehensive cerebrovascular evaluation and the stringent follow-up with repeated standardized measurements. Our MES records were independently assessed by two experienced observers to increase data reproducibility, and the interrater reliability was substantial. However, the study also has some limitations. The sample size is small, reducing the possibility of subgroup analyses. Due to the small sample size and non-consecutive patient enrolment, the prevalence of vulnerable plaque features in our study may not be generalizable to all patients with carotid artery disease. Still, we believe that the temporal changes described in our study are generalizable to the target population. With respect to MES detection, it is possible that some cerebral microemboli may have originated from other sources such as atrial fibrillation, valvular heart disease, or aortic arch atherosclerosis. None of the patients had documented atrial fibrillation on Holter monitoring, but echocardiography and aortic imaging were not performed in all patients. Another limitation is the fact that MRI was not repeated during the follow-up period; hence, clinically silent recurrent infarctions may have been missed.

This study adds to the current knowledge of the course of carotid plaque vulnerability in ischemic stroke. Our future aim is to include ultrasonographic features of plaque vulnerability in vascular risk evaluations to identify patients with high risk and a need for stringent follow-up.

## Conclusions

In patients with acute cerebral ischemia and carotid plaques, we observed a decrease in cerebral microembolization during 6 months of follow-up, indicating plaque stabilization over time. There were no significant changes in IPN during this period.

## Data Availability Statement

The raw data supporting the conclusions of this article will be made available by the authors, without undue reservation.

## Ethics Statement

The studies involving human participants were reviewed and approved by The Regional Committee for Medical and Health Research Ethics of Western Norway (REC West). The patients/participants provided their written informed consent to participate in this study.

## Author Contributions

AF and LT conceived and designed the study. AF, LT, UW-A, and HN contributed to the collection of data. SA and AF analyzed the data and drafted the manuscript. All authors contributed to the interpretation of the results, critically revised the manuscript, and approved the submitted version for publication.

## Conflict of Interest

The authors declare that the research was conducted in the absence of any commercial or financial relationships that could be construed as a potential conflict of interest.

## Publisher's Note

All claims expressed in this article are solely those of the authors and do not necessarily represent those of their affiliated organizations, or those of the publisher, the editors and the reviewers. Any product that may be evaluated in this article, or claim that may be made by its manufacturer, is not guaranteed or endorsed by the publisher.
